# The importance of study design in the application of artificial intelligence methods in medicine

**DOI:** 10.1038/s41746-019-0174-1

**Published:** 2019-10-18

**Authors:** Martin Eklund, Kimmo Kartasalo, Henrik Olsson, Peter Ström

**Affiliations:** 1 Department of Medical Epidemiology and Biostatistics, Karolinska Institutet, Stockholm, Sweden; 2 Faculty of Medicine and Health Technology, Tampere University, Tampere, Finland

**Keywords:** Epidemiology, Prostate cancer

We read the paper Nagpal et al.^1^ with great interest. The authors should be commended on a well-conducted study with an impressive number of pathologists grading the radical prostatectomy samples. We fully agree with the authors that AI will play an integral part in prostate cancer pathology and, indeed, in pathology in general. However, we would like to clarify one methodological detail, which clouds the authors’ claim of “improved Gleason scoring.”

It is an obvious idea to compare the *c*-statistic for the association between longer-term outcomes (such as PSA relapses) and the Gleason grading assigned by the pathologists to the Deep Learning System (DLS). However, the interpretation of this comparison is not straightforward. The challenge is that the original treatment choice is based on the Gleason score assigned by the pathologist. The statistical consequence of this is that the strength of the association can be severely biased. In fact, even if human Gleason grading actually had a stronger association with longer-term outcomes than the AI model, this bias could cause the results to be reversed.

We can draw on a nice analogy from Vickers and Lilja^2^ to illustrate the problem: take a group of 12-year-olds and have them play basketball; height and hand–eye coordination skills will both be the major predictors of ability. Then select out the tallest boys and have them play again; height will no longer discriminate, but the hand–eye coordination skills will still be a major predictor of ability. This does however not imply that being tall is not an advantage for basketball players nor that the hand–eye coordination skills are more predictive than height.

**Fig. 1:**
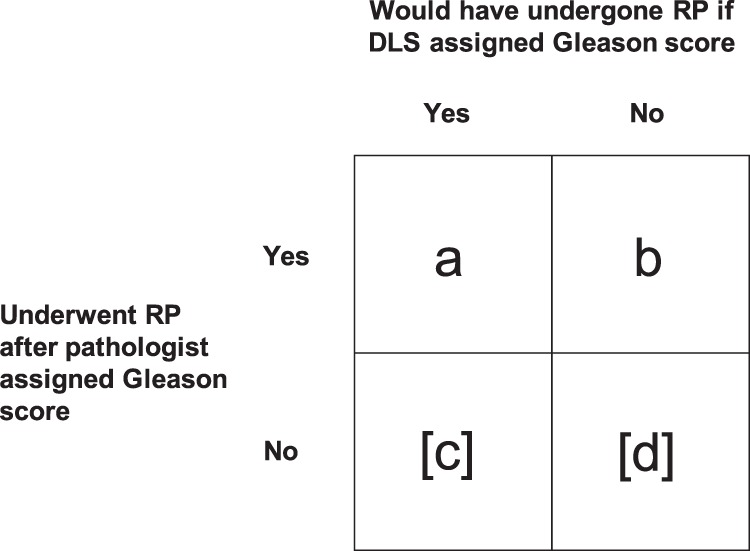
Schematic illustration of the design of the Nagpal et al. study. Data from cells a and b are observed in the study. The fact that data for men who did not undergo radical prostatectomy but would have done so if the treatment decision was based on the DLS as opposed to the pathologists (cell c) can lead to biased results when comparing the pathologists’ Gleason score to the DLS Gleason using longer-term outcomes

More formally, the design of the Nagpal et al. study is schematically illustrated in Fig. 1. Data on men who underwent radical prostatectomy (cell a and b) is observed in the study. Data for men who did not undergo radical prostatectomy but would have done so if the treatment decision was based on the DLS as opposed to the pathologists is however unobserved (cell c, put in brackets to illustrate the fact that it is not observed in the study). The only way to accurately estimate whether the Gleason score assigned by the DLS is in fact better than the Gleason score assigned by the pathologist for prognostication would be to have data from cells *a*, *b*, and *c*,^3^ i.e., to use the DLS for deciding about which patients should undergo radical prostatectomies and then prospectively follow them to collect longer-term follow-up data. The story gets even more complicated if we also consider the fact that the surgeon often is more radical in cases with higher Gleason grades to avoid positive resection margins, and that adjuvant hormonal therapy is administered based on the Gleason score assigned by the pathologist. It should be noted that this problem is not limited to the Nagpal et al. study; it occurs in any study where inclusion in the study cohort is conditional on an existing decision rule and we want to compare the existing decision rule with a new rule.

There is currently a lot of hype related to the application of AI to health care problems. For AI to live up to this hype, it is critical that it is coupled with sound classical experimental designs and scientific methodology.
